# The Broad Spectrum and Continuing Needs of Women’s Health

**DOI:** 10.3390/ijerph19031446

**Published:** 2022-01-27

**Authors:** Colin Binns, Mi Kyung Lee, Lyn Wren

**Affiliations:** 1 School of Population Health, Curtin University, Bentley 6102, Australia; 2 College of Science, Health, Engineering and Education, Murdoch University, Perth 6150, Australia; M.K.Lee@murdoch.edu.au; 3 Quinns Mindarie Super Clinic, Perth 6030, Australia; lwren@qmsclinic.com.au

The Women’s Health section of the *IJERPH* has published almost 700 papers in the past three years, reflecting its importance in public health. This Special Issue, “Feature Papers in Women’s Health”, highlights a selection of topics illustrating the diversity of issues that affect the wellbeing of women. Improving and sustaining the health of women is not only important for the present generation but also to preserve the health of generations to come. The development of modern, scientific medicine over the past two centuries has made great progress in finding solutions to infectious diseases and poor nutrition. However, women face specific risks from issues related to gender and to the effects of events beyond their control. Such events include sex trafficking and intimate partner violence. The continuing exploitation of women in sex trafficking has a devastating effect on their health. Women are also frequently the victims of violence, including from their partners and often more severely during times of conflict and stress. The incidence of domestic violence has increased during the COVID-19 pandemic. 

The COVID-19 pandemic has shown that human society remains vulnerable to new infections and that public health should never become complacent. While mortality rates from infections have generally declined during the 20th century, food, water and vector-borne diseases and chronic diseases have provided an increasing challenge. Now, the world faces what may become the greatest public health challenge it has ever faced—the challenge of climate change. 

A wealth of research in recent years has confirmed the relationship between climate change and human health. There will be increases in zoonoses and food-, water- and vector-borne diseases and mental health issues. Poverty, food insecurity and malnutrition, living in remote and isolated areas and restrictions imposed by social norms all tend to exacerbate the gender-related effects of climate change. Climate change affects reproductive health through changes in extreme weather events, food shortages and malnutrition and increased air pollution. These factors increase the risk of preterm birth, low birthweight, stillbirths and consequent impacts on brain development. In most countries, and particularly in low- and middle-income countries (LMICs), the predetermined social roles of women continue. The WHO noted that everyone is harmed by the impacts of climate change, with harms falling disproportionately on disadvantaged population groups, including women and girls, indigenous communities, people in crisis, displaced people and the economically disadvantaged. The stresses placed on society by the COVID-19 pandemic may be a foretaste of the impact of climate change, even in higher-income societies [1]. 

The increase in environmental pollution being experienced worldwide will impact women’s fertility. Maintaining and improving breastfeeding rates of infants is an important strategy for modifying the impact of climate change on infants, and this may benefit from the application of new communication technologies [2,3]. Sometimes, pollutants may be found in breastmilk, and efforts need be made to continue to reduce environmental pollution, but breastmilk is still the safest and most beneficial way to feed all infants [4]. Cultural pressures, however, continue to subjugate women’s management of their reproductive lives. This is seen in many cultures where girls are breastfed for shorter periods than boys, creating future nutrition and health outcome disparities. This may be due to the pressure to cease lactational amenorrhoea and conceive a male child. 

The COVID-19 pandemic has affected all segments of the population, with males generally having a higher mortality rate than females, but women may experience higher levels of long-term morbidity. In the early months of the pandemic, when it was not known whether COVID-19 could be commonly transmitted to infants via breastmilk, infants were frequently separated from COVID-19-positive mothers [5]. This resulted in a substantial decline in the breastfeeding of infants with all of the negative flow-on effects on the infant’s future life. A substantial proportion of COVID cases, probably about one third, have long-term symptoms, and the majority of these cases will be in women. These women will likely encounter discrimination in their quest for treatment.

The reproductive years of life and childbirth are a time of additional morbidity. In recent decades, the incidence of caesarean section has increased worldwide, with often little or no clinical justification. Iran is one country that has high rates of caesarean section, with many being performed due to the preferences of the parents or the health care provider [6]. This is not without risk to the mother and the infant, who may have additional risks including infections, hysterectomy, anaesthetic complications, anaemia and the costs of the intervention and longer hospital stays. For the infant, there are adverse infant feeding practices associated with caesarean section, including the increased use of infant formula and the reduced duration of breastfeeding with subsequent risk of poor health outcomes [6,7,8]. 

Anaemia is one of the most common nutritional problems for women, present in around one third and in 40% or more during pregnancy. Anaemia is an established risk factor of an increase in several chronic diseases, including cardiovascular diseases and cerebrovascular diseases [9]. Using the extensive national insurance database of Taiwan, Sui and colleagues found that anaemia is a risk factor for haemorrhagic and ischemic stroke in females of reproductive age [9]. This highlights the importance of nutrition in preventing anaemia in women. 

The development of the vaccine against human papilloma virus (HPV) was a significant advancement in the prevention of cervical cancer, which is the third most common cancer in women globally and an important cause of penile carcinoma in men [10]. The vaccine was initially targeted towards girls and now includes boys when they reach the age of 11 or 12. In a systematic review of intervention studies, Acampora et al. have suggested that because of the value of this intervention, integrated approaches, including personalized reminders, etc., information and education activities should be directed towards both adolescents and their parents [10]. There is a need to continually improve the health literacy of women, particularly in LMICs, to help achieve good health during pregnancy and the reproductive years [11]. 

Women’s health will remain a significant issue for health services and public health. As highlighted by Ochola, there are many neglected tropical diseases that raise significant gender issues [12]. This journal will continue to highlight the importance of women’s health for this generation and the next generations of infants and girls. The COVID-19 pandemic has increased pressure on all segments of society through its impact on the march towards universal health access and pressures on food and potable water supplies. However, these issues will disproportionately effect women, and an upcoming Special Issue of the *IJERPH* will focus on COVID and gender issues.

## Data Availability

Not applicable.
